# The product of trunk muscle area and density on the CT image is a good indicator of energy expenditure in patients with or at risk for COPD

**DOI:** 10.1186/s12931-021-01621-2

**Published:** 2021-01-15

**Authors:** Toru Shirahata, Hideaki Sato, Sanehiro Yogi, Kaiji Inoue, Mamoru Niitsu, Tomoe Akagami, Machika Soma, Tomohiko Mio, Makoto Nagata, Satoshi Nakae, Yuki Nishida, Shigeho Tanaka, Fuminori Katsukawa, Hidetoshi Nakamura

**Affiliations:** 1 Department of Respiratory Medicine, Saitama Medical University, 38 Morohongo, Moroyama-machi, Iruma-gun, Saitama, 350-0495 Japan; 2 Department of Radiology, Saitama Medical University, Saitama, Japan; 3 Department of Nutrition and Metabolism, National Institute of Health and Nutrition, National Institutes of Biomedical Innovation, Health and Nutrition, Tokyo, Japan; 4 Graduate School of Engineering Science, Osaka University, Osaka, Japan; 5 Faculty of Nutrition, Kagawa Nutrition University, Saitama, Japan; 6 Sports Medicine Research Center, Keio University, Yokohama, Japan

**Keywords:** Chronic obstructive pulmonary disease, Computed tomography, Physical activity level, Trunk muscle, Energy expenditure

## Abstract

**Background:**

Physical inactivity due to cachexia and muscle wasting is well recognized as a sign of poor prognosis in chronic obstructive pulmonary disease (COPD). However, there have been no reports on the relationship between trunk muscle measurements and energy expenditure parameters, such as the total energy expenditure (TEE) and physical activity level (PAL), in COPD. In this study, we investigated the associations of computed tomography (CT)-derived muscle area and density measurements with clinical parameters, including TEE and PAL, in patients with or at risk for COPD, and examined whether these muscle measurements serve as an indicator of TEE and PAL.

**Methods:**

The study population consisted of 36 male patients with (n = 28, stage 1–4) and at risk for (n = 8) COPD aged over 50 years. TEE was measured by the doubly labeled water method, and PAL was calculated as the TEE/basal metabolic rate estimated by the indirect method. The cross-sectional areas and densities of the pectoralis muscles, rectus abdominis muscles, and erector spinae muscles were measured. We evaluated the relationship between these muscle measurements and clinical outcomes, including body composition, lung function, muscle strength, TEE, and PAL.

**Results:**

All the muscle areas were significantly associated with TEE, severity of emphysema, and body composition indices such as body mass index, fat-free mass, and trunk muscle mass. All trunk muscle densities were correlated with PAL. The product of the rectus abdominis muscle area and density showed the highest association with TEE (r = 0.732) and PAL (r = 0.578). Several trunk muscle measurements showed significant correlations with maximal inspiratory and expiratory pressures, indicating their roles in respiration.

**Conclusions:**

CT-derived measurements for trunk muscles are helpful in evaluating physical status and function in patients with or at risk for COPD. Particularly, trunk muscle evaluation may be a useful marker reflecting TEE and PAL.

## Background

Chronic obstructive pulmonary disease (COPD) is one of the most common chronic respiratory diseases characterized by persistent airflow limitation [1] and is associated with extrapulmonary comorbidities such as cardiovascular diseases, metabolic diseases, depression, osteoporosis, and skeletal muscle dysfunction [1–3].

Approximately 20% of patients with COPD experience weight loss due to inadequate energy intake, increased basal metabolic rate (BMR), and/or increased activity energy expenditure [4, 5]. Additionally, low body weight (BW) and low body mass index (BMI) or fat-free mass (FFM) index are recognized as strong predictors of reduced performance, more frequent exacerbations, decreased quality of life, and increased mortality [6–9]. Waschki et al. investigated the prognostic factors in COPD patients by Cox proportional hazard analysis and found that physical activity was the strongest prognostic factor [10]. Thus, increasing physical activity is expected to improve prognosis, and measurement of energy expenditure is considered to be very important to assess physical activity levels (PALs). In this respect, a number of studies on the energy expenditure of COPD patients have been documented. Among these, only a few studies have utilized the doubly labeled water (DLW) method for determining the total energy expenditure (TEE) in COPD patients [4, 11–13], while others used different motion sensors [11, 14, 15]. The DLW method is the gold standard for TEE assessment because it is the most accurate method to measure total energy expenditure in daily life, which is essential to determine the energy requirement and avoid weight loss in COPD patients. However, this method is quite demanding since it requires significant technical expertise for implementation and analysis and involves high costs and thorough patient compliance to facilitate collection of urine samples. Therefore, studies using the DLW method have been limited to small-sized populations.

Skeletal muscle assessments in COPD have been recently gaining attention because they reflect the severity of COPD more accurately than BMI; since loss of skeletal muscle is not always accompanied by a corresponding loss in adipose tissue, BMI may underestimate muscle wasting [16–18]. Thus, BMI may not accurately characterize the body composition changes during the course of COPD. In contrast, measurement of the cross-sectional area of skeletal muscles on single-slice axial computed tomography (CT) images is an alternative method to assess body composition, including local skeletal muscles [19–23]. Evaluation of skeletal muscles on CT images includes measurement of muscle area and density. In previous studies, the CT cross-sectional areas of the pectoralis muscle (PM), intercostal muscles, erector spinae muscle (ESM), and midthigh muscles have been reported to be associated with FFM, symptoms, disease severity, and prognosis in patients with COPD [19, 22–26]. Although very few studies have attempted CT-derived measurement of muscle density, this approach may indicate muscle quality and decreased physical function [21, 23, 26]. Low muscle density reflects a reduction in muscle contractile units along with fatty replacement [27, 28], which was associated with aging, deconditioning, and disuse in the general population [29, 30]. Since these factors are characteristics of COPD patients, it is conceivable that low muscle density may affect physical inactivity in patients with COPD. However, no previous reports have assessed the relationship between PAL and skeletal muscle mass (SMM) and density in this population.

This study aimed to clarify the associations of the CT measurements of trunk muscle with clinical parameters including lung function, muscle strength, quality of life score, TEE, and PAL in patients with or at risk for COPD. we hypothesized that the product of area and density would be the greatest indicator because both lower mass and lower density contribute to disease severity [23], and the trunk muscle mass and density may serve as an indicator of energy expenditure and physical activity. Reduction in trunk muscle mass in patients with COPD may occur heterogeneously due to the respective muscles’ roles in physiological functions. Therefore, we investigated the area and density of three muscles, namely the PM (PMA/PMD), rectus abdominis muscle (RAM; RAMA/RAMD), and ESM (ESMA/ESMD), which can be assessed without additional radiation exposure or cost. Although PMA and ESMA in COPD patients are correlated with physiological parameters, symptoms, and prognosis [22], there have been no reports on the relationship between RAM and COPD-related traits.

## Methods

### Patients and study design

Patients with COPD (n = 28) and those at risk for COPD (n = 9) were enrolled in this study between June 2017 and February 2018 at the Saitama Medical University Hospital. All patients with COPD [the Global Initiative for Chronic Obstructive Lung Disease (GOLD) 1, 2, 3, 4] and at risk for COPD (GOLD 0) were clinically stable and free of exacerbations for 4 weeks prior to recruitment. COPD was diagnosed in accordance with the GOLD guideline 2020 [31]. All GOLD 0 patients had chronic respiratory symptoms including cough, sputum, or dyspnea on exertion and a ≥ 10 pack year smoking history in the absence of airflow limitation (FEV1/FVC ≥ 0.7) after inhalation of bronchodilators. Since they had similar COPD assessment test (CAT) score and low-attenuation area (LAA%) to those of the COPD patients (9.6 ± 4.0 vs. 10.2 ± 6.7, and 9.4 ± 8.6 vs. 15.1 ± 14.6, respectively) and most of them (6 patients) improved their symptoms by administration of inhaled bronchodilators, we included the GOLD 0 patients in the total population in the present study. One patient at GOLD 0 discontinued the study due to acute bronchitis, and the data from the remaining 36 participants were used for analysis. Exclusion criteria were infectious diseases, diabetes mellitus with medication, dysphagia, or other serious diseases that would interfere with movement; treatment with drugs affecting energy expenditure (thyroid hormone, beta-blocker, glucagon-like peptide-1 receptor agonist) or water balance (sodium-glucose cotransporter-2 blocker); or weight loss more than 5% of the BW during the previous 3 months.

All patients underwent anthropometric measurements, blood test, pulmonary function tests, 6-min walking distance (6MWD) test, health-related quality-of-life questionnaire assessment, COPD assessment test (CAT), modified Medical Research Council (mMRC) dyspnea scale assessment, and chest CT. BW was measured to the nearest 0.1 kg using a digital scale with patients wearing light clothing. The height of each patient was measured to the nearest 0.1 cm using a horizontal headboard with an attached wall-mounted metric scale. BMI was then calculated using these measurements (BW in kg/height in m^2^). FFM and SMM were measured using a bioelectrical impedance analyzer (BIA), SFB7 (ImpediMed, Queensland, Australia). This study was approved by the Institutional Review Board of Saitama Medical University Hospital (No.16–003-1), Keio University (Protocol No. 2015–03) and National Institutes of Biomedical Innovation, Health and Nutrition (Protocol No. 29). Written informed consent was obtained from each participant.

### Pulmonary function testing

After bronchodilator inhalation, spirometry was performed by trained and certified spirometry technicians using a FUDAC-7 instrument (Fukuda Denshi Co., Ltd., Tokyo, Japan). Lung volume subdivisions and diffusing capacity of carbon monoxide (DLco) were measured in all subjects. As indices of respiratory muscle strength, maximal expiratory pressure (PEmax) and maximal inspiratory pressure (PImax) were measured with a spirometer AutospiroAS-507 (Minato Medical Science, Co., Ltd., Osaka, Japan). The predicted pulmonary function values were calculated on the basis of the Japanese Respiratory Society guidelines [32].

### CT scan acquisition and analysis

Chest CT scans (SOMATOM Emotion 16, Siemens Healthcare, Erlangen, Germany) were obtained with the following parameters: collimation, 16 × 1.2 mm; rotation time, 600 ms/rot; pitch factor, 1.05; peak, 130 kV; and automatic exposure control (Quality Reference mAs 100). Routine calibration of the CT scanner was performed using air and water phantoms. For quantitative analysis of pulmonary emphysema and muscles, chest CT images were reconstructed using the mediastinal setting (reconstruction kernel: B41s medium +) with 1.5-mm thickness. The cross-sectional area and density of the PM from a single axial slice of chest CT at the top of the aortic arch (PMA/PMD), the RAM at the first lumbar vertebra (RAMA/RAMD), and the ESM at the level of Th12 lumbar vertebra (ESMA/ESMD) were measured.

**Fig. 1:**
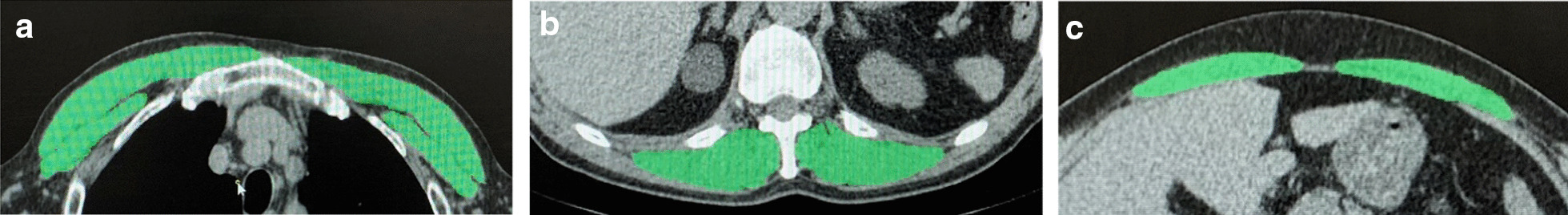
Representative computed tomographic images used to measure the cross-sectional areas of the trunk muscles. The pectoralis muscle (PM) (**a**), erector spinae muscle (ESM) (**b**), and the rectus abdominis muscle (RAM) (**c**) are shown in green

We used a SYNAPSE VINCENT volume analyzer software (FUJIFILM Medical Co., Ltd., Tokyo, Japan) for all analyses. Details regarding muscle measurements are provided below; after imaging, the percentage of the low-attenuation area (LAA%) was determined using a cutoff value of -950 Hounsfield units (HU). Then, the left and right muscles were identified and manually shaded using a predefined attenuation range of -50 and 90 HU. Muscle areas on both sides were measured, and the muscle area was presented as the sum of the right and left muscles, and average muscle density was defined as the mean attenuation within the drawn regions (Fig. 1a-c).

### Measurement of BMR and TEE

#### Measurement of BMR

BMR was measured by indirect calorimetry (Quark BMR; COSMED, Rome, Italy). Before BMR measurements, the subjects were allowed to ingest only water for 12 h. The test was performed between 08:30 and 10:00. The Quark BMR measures the amount of oxygen consumed and carbon dioxide exhaled, and calculates the BMR using the modified Weir Eq. [33]. Before measurement, the procedure was explained to the subjects, who had comfortably rested on a bed for 30 min. A steady state was achieved for more than 5 min by the Quark BMR after 10–15 min of breathing while the subjects lay awake in the supine position.

#### Measurement of TEE by the DLW method

TEE of each patient was measured over 13–15 days by the DLW method. The patients were instructed to behave as usual during the study period. The DLW protocol details are given elsewhere [34]. In brief, on the morning of day 1 (visit 1), after the collection of background blood and urine samples, subjects took an oral dose of 0.1 g ^2^H_2_O and 2.0 g H_2_^18^O (Taiyo Nippon Sanso, Tokyo, Japan) per kilogram of estimated total body water. Post-dose blood sample was collected at only 4 h while post-dose urine samples were collected at 2, 3, and 4 h. Further samples were collected on the mornings of days 13–15 at visit 2 (once for blood and twice for urine at a 1-h interval). In the collected samples, the representative TEE value was calculated by determining the average TEEs obtained from both samples at background, 4 h post-ingestion, and at visit 2 after emptying the urinary bladder. Isotope analyses of the blood and urine samples were performed at ESTech Kyoto (Kyoto, Japan) in duplicate using an isotope-ratio mass spectrometer (Hydra 20–20 Stable Isotope Mass Spectrometer; Sercon, Crewe, UK). The DLW method provides an average TEE value per day during the period of assessment. The PAL was calculated using the following equation: PAL =  (TEE estimated by DLW method)/ (BMR measured by indirect calorimetry) [35].

### Statistical analysis

Data pertaining to the continuous variables are expressed as mean ± standard deviation unless otherwise specified. All statistical analyses were performed using JMP version 14 software (SAS Institute Inc., Cary, NC, USA). A bivariate correlation analysis was performed between muscles and clinical parameters such as anthropometric, pulmonary function, and emphysema severity measurements. Multiple regression analyses were used to adjust for confounders, age and height, to explore the clinical importance of the relationship between muscle indices and energy expenditure. Multiple comparison was performed with the Tukey–Kramer test. P values less than 0.05 were considered statistically significant. To assess the reliability of statistically significant measurements of the muscle areas and densities, intra-class correlation coefficients (ICC) were calculated to determine relative reliability. ICC (1, 1) was calculated to examine the intra-rater reliability, and ICC (2, 1) was used to determine inter-rater reliability.

## Results

### Patient characteristics

The baseline characteristics of the study subjects are shown in Table 1. The patients showed a wide spectrum of disease severity in terms of lung function (FEV1 from 27.1% predicted to 110.3% predicted).

**Table 1:** Patient characteristics

Variable	Data (range)
Total number of patients	36
Sex, M/F	36/0
Age, year	70.3 ± 5.8 (52–78)
Height, m	1.65 ± 0.1 (1.53–1.77)
Weight, kg	60.2 ± 10.1 (39.8–81.0)
BMI, kg/m^2^	21.9 ± 3.2 (15.2–30.1)
Smoking history, pack-years	56.5 ± 23.3 (25–120)
GOLD 0/1/2/3/4	8/6/14/6/2
FEV1, L	1.89 ± 0.7 (0.7–3.3)
FEV1, %predicted	69.4 ± 24.4 (27.1–110.3)
FEV1/FVC, %	55.4 ± 17.4 (27.0–94.1)
DLco/V_A_, %predicted	74.0 ± 27.4 (28.9–127.0)
LAA% less than -950 HU, %	13.9 ± 13.6 (0.1–52.0)
mMRC dyspnea scale score, 0/1/2/3/4	14/13/6/3/0
6MWD, m	434.9 ± 94.6 (275–750)
CAT score, n	10.1 ± 6.1 (1–29)
FFM, kg	45.5 ± 7.4 (30.0–59.2)
SMM, kg	24.9 ± 3.4 (18.2–30.2)
HGS, kg	33.9 ± 7.0 (22.1–46.9)
CC, cm	33.3 ± 3.1 (26.9–38.8)
PEmax, cmH_2_O	75.4 ± 22.6 (36.1–143.2)
PImax, cmH_2_O	63.6 ± 21.2 (27.3–110.3)
TEE, kcal/day	2273 ± 445.4 (1265–3453)
BMR, kcal/day	1262 ± 179.9 (863.0–1694)
PAL	1.80 ± 0.20 (1.36–2.16)
PMA, cm^2^	30.8 ± 5.8 (18.5–46.5)
PMD, HU	32.6 ± 7.0 (14.6–43.5)
RAMA, cm^2^	8.7 ± 1.7 (5.5–12.8)
RAMD, HU	26.5 ± 6.8 (12.5–39.0)
ESMA, cm^2^	26.2 ± 5.0 (12.6–36.6)
ESMD, HU	36.4 ± 9.7 (-3.2–54.1)

*BMI* body mass index, *GOLD* Global Initiative for Chronic Obstructive Lung Disease, *FEV* forced expiratory volume, *VC* vital capacity, *DL*_*CO*_*/V*_*A*_ diffusing capacity of carbon monoxide/alveolar volume, *LAA*% percentage of the lung field occupied by low-attenuation areas, *mMRC* modified Medical Research Council, *CAT* COPD assessment test, *FFM* fat-free mass, *SMM* skeletal-muscle mass, *HGS* hand-grip strength, *CC* calf circumference, *PEmax* maximal expiratory pressure, *PImax* maximal inspiratory pressure, *TEE* total energy expenditure, *BMR* basal metabolic rate, *PAL* physical activity level, *PMA* pectoralis muscle area, *PMD* pectoralis muscle density, *RAMA* rectus abdominis muscle area, *RAMD* rectus abdominis muscle density, *ESMA* erector spinae muscle area, *ESMD* erector spinae muscle density, *HU* Hounsfield unit

### Distribution of the three muscle areas and densities

Distributions of muscle areas (in cm^2^) and densities (in HU) by GOLD grade in cross-sectional analysis are shown in Table 2. Although muscle areas, densities, and their products in patients with GOLD 3, 4 tended to be lower than those in the other groups, all of the indices were not significantly different among the GOLD grades. When the areas were normalized with height^2^, the trends were similar to those of PMA, RAMA, and ESMA.

**Table 2:** Distributions of patient’s characteristics including muscle areas (in cm^2^) and densities (in HU) by GOLD grade

	GOLD 0	GOLD 1	GOLD 2	GOLD 3, 4
N	8	6	14	8
Age, years	70.3 ± 7.1	68.8 ± 8.9	71.36 ± 4.4	69.6 ± 4.5
Height, m	1.65 ± 0.08	1.65 ± 0.06	1.65 ± 0.05	1.66 ± 0.05
Weight, kg	59.1 ± 13.0	60.8 ± 6.6	63.6 ± 9.6	54.8 ± 9.2
BMI, kg/m^2^	21.2 ± 3.7	21.8 ± 1.3	23.5 ± 3.0^※^	19.7 ± 3.2
CAT score, n	9.6 ± 4.0	10.0 ± 7.0	7.8 ± 5.6	14.6 ± 6.7
LAA%	9.4 ± 8.6^#^	8.3 ± 5.6^#^	9.2 ± 8.2^#^	30.7 ± 17.0
PMA	30.2 ± 6.8	30.1 ± 3.0	32.7 ± 6.3	28.6 ± 5.1
PMD	32.9 ± 8.6	34.1 ± 6.5	33.9 ± 6.7	28.9 ± 5.8
PM product	1030.2 ± 425.7	1035.4 ± 274.8	1133.1 ± 391.3	838.8 ± 245.5
RAMA	8.5 ± 2.1	8.7 ± 1.0	9.1 ± 1.6	8.1 ± 1.7
RAMD	25.5 ± 6.0	29.5 ± 7.7	27.5 ± 7.2	23.7 ± 6.1
RAM product	222.7 ± 98.0	260.0 ± 81.8	257.5 ± 93.1	198.9 ± 85.6
ESMA	25.3 ± 5.4	26.5 ± 6.1	27.6 ± 4.9	24.3 ± 3.9
ESMD	34.9 ± 8.6	39.8 ± 2.6	37.2 ± 12.5	33.9 ± 8.9
ESM product	894.6 ± 344.0	1056.5 ± 280.0	1077.0 ± 361.8	843.9 ± 346.3
PMA /Ht^2^	11.0 ± 2.3	11.0 ± 0.8	12.1 ± 2.4	10.4 ± 1.8
RAMA /Ht^2^	3.1 ± 0.8	3.2 ± 0.2	3.4 ± 0.5	3.0 ± 0.7
ESMA /Ht^2^	9.3 ± 1.8	9.7 ± 2.0	10.2 ± 1.7	8.8 ± 1.3

*PMA* pectoralis muscle area, *PMD* pectoralis muscle density, *RAMA* rectus abdominis muscle area, *RAMD* rectus abdominis muscle density, *ESMA* erector spinae muscle area, *ESMD* erector spinae muscle density, *Ht* height

^※^p < 0.05 vs. GOLD3,4

^#^p < 0.01 vs GOLD3,4

The intra and inter- observer reliabilities were determined for the muscle areas and densities in this study by two well-trained pulmonary physicians. The ICC of the intra-observer agreement was 0.994 for the area and 0.984 for the density, respectively. The ICC of the inter-observer reliability was 0.982 for the area and 0.969 for the density, respectively.

### Bivariate correlation analysis

#### Relationship between muscle areas and density in each muscle

**Fig. 2:**
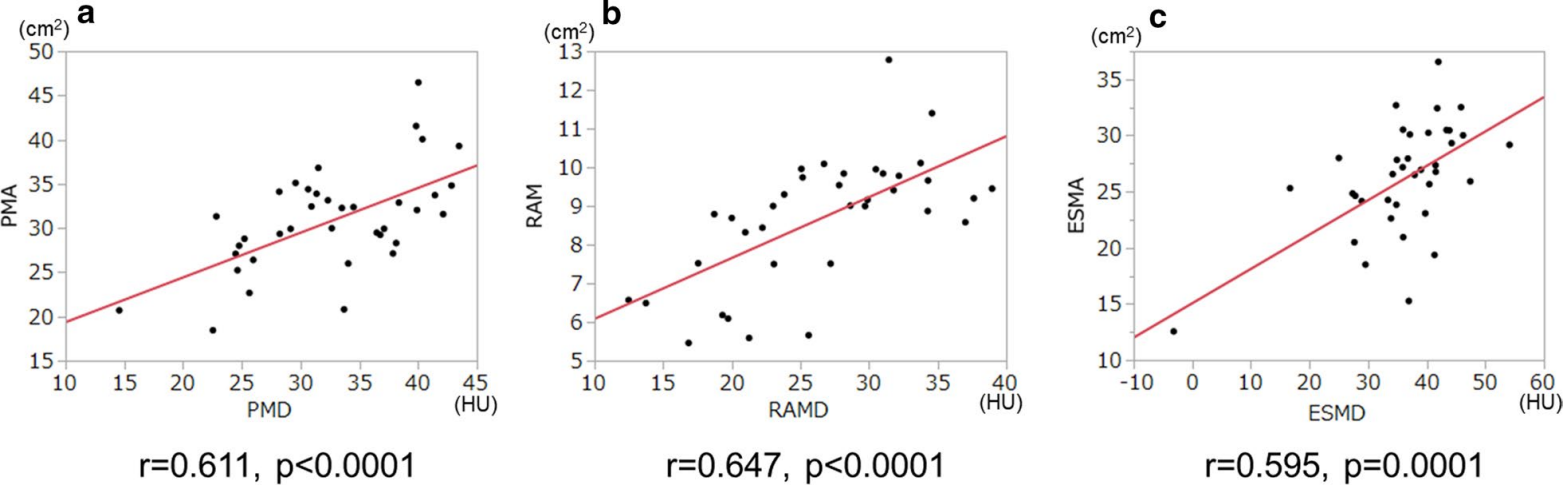
The relationship between area and density of each muscle. Significant associations were observed between the two variables in the pectoralis muscle (**a**), rectus abdominis muscle (**b**), and erector spinae muscle (**c**). *PMA* pectoralis muscle area, *PMD* pectoralis muscle density, *RAMA* rectus abdominis muscle area, *RAMD* rectus abdominis muscle density, *ESMA* erector spinae muscle area, *ESMD* erector spinae muscle density

**Fig. 3:**
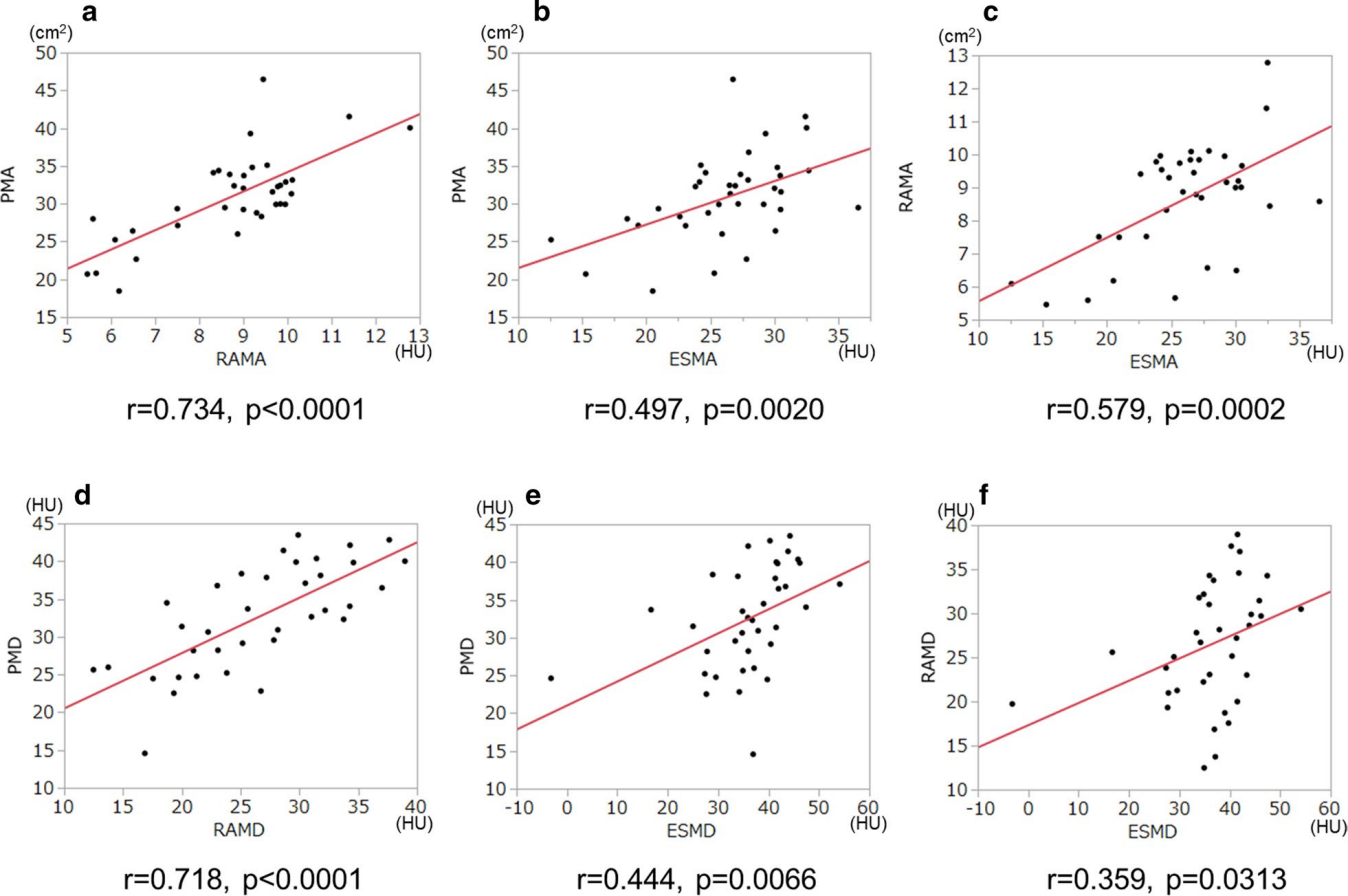
Relationships among muscles (area or density): PMA and RAMA (**a**), PMA and ESMA (**b**), RAMA and ESMA (**c**), PMD and RAMD (**d**), PMD and ESMD (**e**), and RAMD and ESMD (**f**). In these comparisons, both areas and densities showed good correlations between the pectoralis and rectus abdominis muscles (**a**, **d**). *PMA* pectoralis muscle area, *PMD* pectoralis muscle density, *RAMA* rectus abdominis muscle area, *RAMD* rectus abdominis muscle density, *ESMA* erector spinae muscle area, *ESMD* erector spinae muscle density

CT scans of PM, RAM, and ESM revealed significant associations between the area and the density of each muscle (Fig. 2). Good concordance was observed in the muscle areas and densities among the three muscles (Fig. 3).

#### Relationship between clinical traits and muscle area, density, and area–density products

Bivariate analysis of the CT-derived cross-sectional indices of the three muscles and various clinical parameters are shown in Tables 3, 4, and 5. Both indices of airflow limitation, %FEV_1_ and FEV_1_/FVC, did not significantly correlate with any of the trunk muscle measurements (Tables 3, 4, and 5). In contrast, indices of emphysema, %DL_CO_/V_A_ and LAA%, correlated with most of these measurements. When patients at GOLD 0 were excluded, PMD and RAMD were correlated with %FEV_1_ and FEV_1_/FVC (p < 0.05), and PM product and RAM product were correlated with FEV_1_/FVC (p < 0.05).

**Table 3:** Bivariate analysis of the CT-derived cross-sectional indices of the pectoralis muscle and clinical parameters

Variable	PMA		PMD		PM product	
	r	P	r	p	r	p
Age, years	0.226	0.186	0.134	0.435	0.193	0.260
Height, m	0.228	0.181	0.405	0.014	0.329	0.050
Weight, kg	0.569	< 0.001	0.497	0.002	0.557	< 0.001
BMI, kg/m^2^	0.566	< 0.001	0.414	0.012	0.507	0.002
CAT, n	0.374	0.025	0.490	0.779	0.212	0.215
mMRC score, units	0.058	0.736	0.260	0.126	0.191	0.265
6MWD, m	0.246	0.148	0.268	0.114	0.306	0.069
FEV1, % predicted	0.076	0.659	0.289	0.088	0.225	0.186
FEV1/FVC, %	0.019	0.915	0.197	0.249	0.194	0.384
DLco/V_A_, % predicted	0.395	0.017	0.465	0.004	0.512	0.001
LAA%, %	0.350	0.036	0.336	0.045	0.385	0.020
FFM, kg	0.660	< 0.0001	0.572	< 0.001	0.659	< 0.0001
SMM, kg	0.573	< 0.001	0.598	< 0.001	0.628	< 0.0001
HGS, kg	0.491	0.002	0.473	0.004	0.521	0.001
CC, cm	0.595	< 0.001	0.497	0.002	0.572	< 0.001
TEE, kcal/day	0.630	< 0.0001	0.646	< 0.0001	0.680	< 0.0001
BMR, kcal/day	0.506	0.002	0.467	0.004	0.504	0.002
PAL	0.483	0.003	0.551	< 0.001	0.555	< 0.001
PEmax, cmH_2_O	0.313	0.063	0.416	0.012	0.373	0.025
PImax, cmH_2_O	0.096	0.577	0.486	0.003	0.324	0.054

*BMI* body mass index, *CAT* COPD assessment test, *mMRC* modified Medical Research Council scale, *6MWD* 6-min walking distance, *FEV* forced expiratory volume, *DL*_*CO*_*/V*_*A*_ diffusing capacity of carbon monoxide/alveolar volume, *LAA%* percentage of the lung field occupied by low-attenuation areas, *FFM* fat-free mass, *SMM* skeletal-muscle mass, *HGS* hand-grip strength, *CC* calf circumference, *TEE* total energy expenditure, *BMR* basal metabolic rate, *PAL* physical activity level, *PEmax* maximal expiratory pressure, *PImax* maximal inspiratory pressure, *PMA* pectoralis muscle area, *PMD* pectoralis muscle density

**Table 4:** Bivariate analysis of the CT-derived cross-sectional indices of the rectus abdominis muscle and clinical parameters

Variable	RAMA		RAMD		RAM product	
	r	P	r	p	r	p
Age, year	0.150	0.382	0.292	0.084	0.261	0.124
Height, m	0.279	0.100	0.251	0.140	0.277	0.103
Weight, kg	0.569	< 0.001	0.418	0.011	0.518	0.001
BMI, kg/m^2^	0.571	< 0.001	0.380	0.022	0.488	0.003
CAT, n	0.247	0.146	0.127	0.459	0.199	0.246
mMRC score, units	0.037	0.831	0.260	0.881	0.012	0.947
6MWD, m	0.241	0.157	0.262	0.123	0.296	0.079
FEV1, % predicted	0.059	0.731	0.278	0.100	0.201	0.240
FEV1/FVC, %	0.023	0.893	0.182	0.288	0.104	0.545
DLco/V_A_, % predicted	0.238	0.163	0.338	0.044	0.339	0.043
LAA%, %	0.384	0.021	0.296	0.079	0.359	0.031
FFM, kg	0.624	< 0.0001	0.510	0.002	0.612	< 0.0001
SMM, kg	0.542	< 0.001	0.513	0.001	0.575	< 0.001
HGS, kg	0.519	0.001	0.395	0.017	0.503	0.002
CC, cm	0.594	< 0.001	0.507	0.002	0.587	< 0.001
TEE, kcal/day	0.663	< 0.0001	0.679	< 0.0001	0.732	< 0.0001
BMR, kcal/day	0.517	0.001	0.498	0.002	0.538	< 0.001
PAL	0.494	0.002	0.542	< 0.001	0.578	< 0.001
PEmax, cmH_2_O	0.361	0.031	0.361	0.031	0.392	0.018
PImax, cmH_2_O	0.005	0.975	0.133	0.438	0.060	0.726

*BMI* body mass index, *CAT* COPD assessment test, *mMRC* modified Medical Research Council scale, *6MWD* 6-min walking distance, *FEV* forced expiratory volume, *DL*_*CO*_*/V*_*A*_ diffusing capacity of carbon monoxide/alveolar volume, *LAA%* percentage of the lung field occupied by low-attenuation areas, *FFM* fat-free mass, *SMM* skeletal-muscle mass, *HGS* hand-grip strength, *CC* calf circumference, *TEE* total energy expenditure, *BMR* basal metabolic rate, *PAL* physical activity level, *PEmax* maximal expiratory pressure, *PImax* maximal inspiratory pressure, *RAMA* rectus abdominis muscle area, *RAMD* rectus abdominis muscle density

**Table 5:** Bivariate analysis of the CT-derived cross-sectional indices of the erector spinae muscle and clinical parameters

Variable	ESMA		ESMD		ESM product	
	r	P	R	P	r	p
Age, year	0.244	0.152	0.005	0,978	0.189	0.270
Height, m	0.429	0.009	0.203	0.236	0.313	0.063
Weight, kg	0.643	< 0.0001	0.185	0.280	0.439	0.007
BMI, kg/m^2^	0.598	< 0.001	0.182	0.289	0.433	0.008
CAT, n	0.337	0.045	0.081	0.64	0.203	0.235
mMRC score, units	0.196	0.253	0.230	0.178	0.262	0.123
6MWD, m	0.156	0.363	0.190	0.266	0.219	0.199
FEV1, % predicted	0.017	0.923	0.089	0.604	0.066	0.703
FEV1/FVC, %	0.005	0.975	0.140	0.416	0.051	0.768
DLco/V_A_, % predicted	0.401	0.015	0.230	0.176	0.330	0.049
LAA%, %	0.344	0.040	0.154	0.371	0.250	0.141
FFM, kg	0.739	< 0.0001	0.315	0.061	0.591	< 0.001
SMM, kg	0.719	< 0.0001	0.287	0.089	0.567	< 0.001
HGS, kg	0.646	< 0.0001	0.496	0.002	0.678	< 0.0001
CC, cm	0.647	< 0.0001	0.292	0.084	0.512	0.001
TEE, kcal/day	0.620	< 0.0001	0.333	0.047	0.534	< 0.001
BMR, kcal/day	0.614	< 0.0001	0.114	0.509	0.397	0.017
PAL	0.323	0.055	0.419	0.011	0.423	0.010
PEmax, cmH_2_O	0.348	0.038	0.191	0.265	0.300	0.075
PImax, cmH_2_O	0.349	0.037	0.391	0.018	0.429	0.009

*BMI* body mass index, *CAT* COPD assessment test, *mMRC* modified Medical Research Council scale, *6MWD* 6-min walking distance, *FEV* forced expiratory volume, *DL*_*CO*_*/V*_*A*_ diffusing capacity of carbon monoxide/alveolar volume, *LAA%* percentage of the lung field occupied by low-attenuation areas, *FFM* fat-free mass, *SMM* skeletal-muscle mass, *HGS* hand-grip strength, *CC* calf circumference, *TEE* total energy expenditure; *BMR* basal metabolic rate, *PAL* physical activity level, *PEmax* maximal expiratory pressure, *PImax* maximal inspiratory pressure, *ESMA* erector spinae muscle area, *ESMD* erector spinae muscle density

Although only PMA and ESMA were weakly correlated with the CAT score, none of the trunk muscle measurements showed significant correlations with mMRC score or 6MWD. Among body composition indices, BMI was correlated with all nine measurements, but the r values (highest value, 0.570 with PMA) were generally lower than those for FFM (highest value, 0.739 with ESMA) and SMM (highest value, 0.719 with ESMA). The hand-grip strength (highest value, 0.678 with ESM product) and CC (highest value, 0.647 with ESMA) were significantly correlated with all measurements as well.

#### Relationships between energy expenditure and muscle area, density, and area–density products

Most of the muscle indices were well correlated with TEE, BMR, and PAL. The best correlations to TEE, BMR, and PAL were observed for RAM product (r = 0.732), ESMA (r = 0.614), and RAM product (r = 0.578), respectively. After adjustment for age and height, all the same muscle indices were still correlated with TEE and PAL, e. g., the β value of the RAM product for TEE was 0.620 and that for PAL was 0.545, except for ESMD with TEE (Table 6). When adjusted for age, height, and weight, the β value of the RAM product for TEE decreased (β = 0.470, p < 0.0001) while that for PAL increased (β = 0.628, p < 0.001) compared with those for age and height. Similarly, when adjusted for age and BMI, the β value for TEE decreased (β = 0.529, p < 0.001) while the β value for PAL increased (β = 0.664, p < 0.001). TEE and PAL were correlated with most of the muscle measurements even after GOLD 0 patients were excluded (Table 7).

**Table 6:** Results of multiple regression analyses of TEE and PAL, with adjustment for age and height

Variable	TEE		PAL		VIF
	β	p	β	p	
PMA	0.522	< 0.001	0.442	0.008	1.076
PMD	0.508	< 0.001	0.501	0.004	1.201
PM product	0.548	< 0.0001	0.504	0.002	1.124
RAMA	0.545	< 0.0001	0.437	0.009	1.085
RAMD	0.572	< 0.0001	0.517	0.002	1.114
RAM product	0.620	< 0.0001	0.545	0.001	1.111
ESMA	0.456	0.002	0.226	0.221	1.230
ESMD	0.242	0.097	0.370	0.026	1.053
ESM product	0.390	0.007	0.357	0.037	1.112

*TEE* total energy expenditure, *PAL* physical activity level, *PMA* pectoralis muscle area, *PMD* pectoralis muscle density, *RAMA* rectus abdominis muscle area, *RAMD* rectus abdominis muscle density, *ESMA* erector spinae muscle area, *ESMD* erector spinae muscle density

**Table 7:** Bivariate analysis of the CT-derived cross-sectional indices of the trunk muscles and TEE or PAL in the COPD patients (GOLD 1 ~ 3, n = 28)

Variable	TEE		PAL	
	r	p	r	p
PMA	0.559	0.002	0.344	0.073
PMD	0.575	0.001	0.507	0.006
PM product	0.600	0.001	0.455	0.015
RAMA	0.643	< 0.001	0.362	0.059
RAMD	0.672	< 0.0001	0.497	0.007
RAM product	0.713	< 0.0001	0.492	0.008
ESMA	0.556	0.002	0.223	0.253
ESMD	0.425	0.024	0.431	0.022
ESM product	0.538	0.003	0.365	0.056

*TEE* total energy expenditure, *PAL* physical activity level, *PMA* pectoralis muscle area, *PMD* pectoralis muscle density, *RAMA* rectus abdominis muscle area, *RAMD* rectus abdominis muscle density, *ESMA* erector spinae muscle area, *ESMD* erector spinae muscle density

#### Relationships between respiratory muscle strength and muscle area, density, and area–density products

RAMA (r = 0.361), RAMD (r = 0.361), and RAM product (r = 0.392) modestly correlated with PEmax, while ESMA (r = 0.349), ESMD (r = 0.391), and ESM product (r = 0.429) correlated with PImax. As for PM measurements, PMD showed the best correlations with PEmax (r = 0.416) and PImax (r = 0.486) among all comparisons with respiratory muscle strength.

#### Relationship between PAL and the products of muscle area and density

**Fig. 4:**
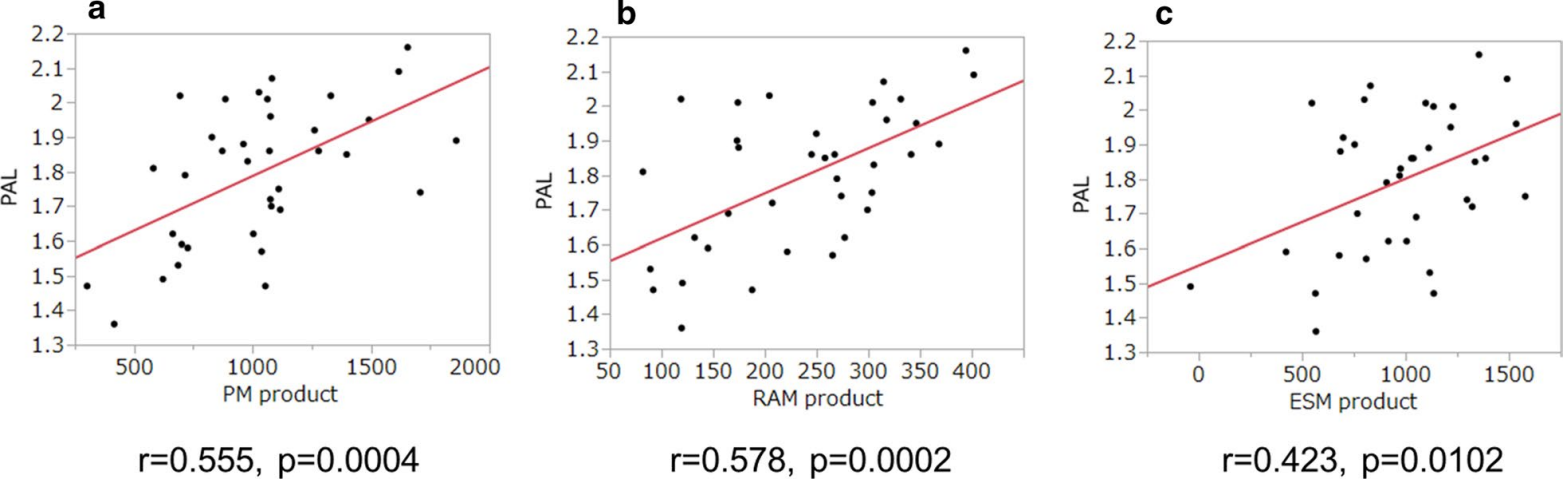
Relationships between PAL and the products of areas and densities of each muscle. PAL shows significant associations with PM product (**a**), RAM product (**b**), and ESM product (**c**). *PM* pectoralis muscle, *RAM* rectus abdominis muscle, *ESMA* erector spinae muscle, *PAL* physical activity level

Relationships between PAL and the products of muscle area and density were presented in Fig. 4. Significant correlations were observed in all three muscles.

### Discussion

To the best of our knowledge, this is the first report to present the relationships between CT-derived trunk muscle measurements and energy expenditure and physical activity evaluated by the DLW method in COPD. Almost all of the areas and densities of trunk muscles such as the PM, RAM, and ESM were significantly associated with TEE and PAL. Additionally, the product of the area and density of the RAM could be the best indicator reflecting TEE (r = 0.732) and PAL (r = 0.578) in patients with and at risk for COPD. These observations suggest a possible role of chest CT-based trunk muscle assessments in evaluating energy expenditure in patients with COPD and the importance of RAM analysis, which has not been reported previously. We also demonstrated the associations of the areas with the densities of the three muscles, and of the areas and densities among the three muscles, and of those with the respiratory muscle strength. Finally, this is the first study to introduce the product of area and density as a promising tool for evaluating the skeletal muscles using CT images.

Chest CT is regularly performed in clinical settings to evaluate emphysema and other causes of acute exacerbation and to determine the development of other respiratory diseases, such as lung cancer. With single-axial CT images, several studies have revealed that skeletal muscle mass, especially PMA, indicates COPD traits [19, 23] and lower PMA affects mortality—even in smokers without airflow limitation [36]. These data are consistent with the finding that PMA in GOLD 0 patients was not different from those who had mild to moderate airflow limitation in our study. The GOLD 0 patients in our study had both a substantial smoking history and respiratory symptoms, and most of them required treatment with bronchodilators. We found no correlation between the trunk muscle measurements and the indices of airflow limitation in the population of GOLD 0 to 4, but there was a correlation in GOLD 1 to 4 patients.

Low FFM and CT-derived muscle areas were reportedly associated with greater impairment in symptoms, activity, and the total St George's Respiratory Questionnaire (SGRQ) score in COPD patients [19, 37, 38]. We showed that the quality of life score as measured by CAT was associated with PMA and ESMA, and any trunk muscle densities were not significantly correlated with these indicators, yielding the same conclusion that COPD-related symptoms may be affected by muscle area rather than muscle density, as corroborated by a previous study [23], although the reasons were unknown.

We used the DLW method to measure energy expenditure in patients with COPD, which is considered to be our study’s strength. All three muscle areas were associated with TEE and BMR in patients with or at risk for COPD. PMA and RAMA were correlated with PAL, but the difference did not reach statistical significance between ESMA and PAL (r = 0.055). The reason for this is unclear, but there were several patients who had reduced PMA and preserved ESMA in our study (Fig. 3b). There were better correlations between PM and RAM in both areas and densities (Fig. 3a, d) than those between ESM and PM or RAM (Fig. 3b, c, e, f). Besides, a previous study reported that ESMA reflected symptoms, pulmonary functions, and the prognosis of COPD more than PMA [22], which was different from our data, and may partly be explained by the differences in the severities of airflow limitation (mean %FEV1: 69.4 vs. 57.6) and emphysema (mean LAA%: 13.9 vs. 33.2).

This study also found that lower muscle density evaluated by CT images was significantly associated with declines in physical activity. Although there were modest associations between the muscle areas and densities in the PM, RAM, and ESM (r = 0.611, r = 0.647, and r = 0.595, respectively, in Fig. 2), the densities of all three muscles showed a stronger correlation with PAL than the muscle areas did (Tables 3, 4, 5, 6, 7), suggesting that not only muscle volume but also muscle quality would be essential for maintenance of physical activity in COPD. The evaluation of the RAM in energy expenditure showed similar trends to the PM in both area and density, indicating that assessment of abdominal muscles would also be a useful indicator for physical status, including PAL, in patients with COPD. However, ESMD was less correlated with PAL. It should, however, be noted that the best correlations with FFM (r = 0.739), SMM (r = 0.719), and CC (r = 0.647) were observed for ESMA, indicating that this parameter could be used to estimate muscle mass and strength in the whole body.

In addition to these findings, we demonstrated that the products of trunk muscle area and density were significantly associated with PAL in this study (Fig. 4). These significant correlations were greater than those with area alone or density alone, and other clinical parameters, suggesting that the product was one of the best predictors of PAL in COPD patients. Bak et al. reported that higher PMA and PMD in patients with COPD indicated increased lung function, and decreased PMA and PMD were significantly associated with emphysema, suggesting that COPD patients with emphysema exhibited declines in muscle mass and quality [23]. Moreover, we observed associations between muscle area and density for all three muscles (Fig. 2). These results indicate that the product of the trunk muscle areas and densities would be a useful indicator of physical status and functions in COPD.

PM usually functions as an inspiratory assist muscle, but it has been reported that the pectoralis major may also act as an expiratory assist muscle [39]. RAM is thought to act as an auxiliary muscle during expiration with the elevation of the diaphragm. ESM usually acts as an antigravity muscle, but is believed to act as a respiratory assist muscle in situations where movement of the diaphragm is restricted [40]. Our results indicated the indices of RAM modestly correlated with PEmax, while those of ESM correlated with PImax. Additionally, PMD showed the best correlations with PEmax (r = 0.416) and PImax (r = 0.486). These findings corresponded to the results of previous reports and suggested the usefulness of the evaluation of trunk muscle density, area, and product as an alternative approach.

This study has several limitations. First, healthy subjects were not included. Second, the sample size was relatively small with 36 cases because of difficulties in the DLW method as previously described. Further studies that include a larger number of participants may enhance the generalizability of our results. Third, female patients were not included since there were several reports that sex differences could exist in skeletal muscle manifestations [23, 36]. Thus, it may be reasonable to unify the gender in this study. Finally, there are no standard methods for measuring the skeletal muscle area and density. We performed manual segmentation of the muscles after the application of a density mask between -50 and + 90 HU and chose single slices of PMA and ESMA according to the previous reports [19, 22, 23] since a previous study showed that single-slice measurements of the skeletal muscle were strongly correlated with the average measurements of three consecutive slices [22].

## Conclusions

The area and density of PM, RAM, and ESM in patients with or at risk for COPD obtained from a single-slice axial chest CT image were associated with the clinical COPD traits, and the product of area and density in the PM and RAM may be a promising indicator of energy expenditure and physical activity in male COPD patients.

## Data Availability

The datasets used and/or analysed during the current study are available from the corresponding author on reasonable request.

## Abbreviations

COPD: Chronic obstructive pulmonary disease
BMR: Basal metabolic rate
BW: Body weight
BMI: Body mass index
FFM: Fat-free mass
SMM: Skeletal muscle mass
DLW: Doubly labeled water
PAL: Physical activity level
TEE: Total energy expenditure
CT: Computed tomography
PM: Pectoralis muscle
ESM: Erector spinae muscle
PMA: PM area
PMD: PM density
RAM: Rectus abdominis muscle
RAMA: RM area
RAMD: RM density
ESMA: ESM area
ESMD: ESM density
GOLD: Global Initiative for Chronic Obstructive Lung Disease
FEV_1_: Forced expiratory volume during the first second
FVC: Forced vital capacity
6MWD: 6-Min walking distance
CAT: COPD assessment test
mMRC: Modified Medical Research Council
BIA: Bioelectrical impedance analyzer
DLco: Diffusing capacity of carbon monoxide
LAA%: Low-attenuation area
HU: Hounsfield units
PEmax: Maximal expiratory pressure
PImax: Maximal inspiratory pressure
SGRQ: St George's Respiratory Questionnaire
